# Integrated Analysis Reveals S100a8/a9 Regulates Autophagy and Apoptosis through the MAPK and PI3K-AKT Signaling Pathway in the Early Stage of Myocardial Infarction

**DOI:** 10.3390/cells11121911

**Published:** 2022-06-13

**Authors:** Weijue Yi, Rongli Zhu, Xiuyang Hou, Fengmin Wu, Rui Feng

**Affiliations:** Department of Pharmaceutical Toxicology, School of Pharmacy, China Medical University, No. 77 Puhe Road, Shenyang North New Area, Shenyang 110122, China; eve_yiweijue@163.com (W.Y.); julyft23@163.com (R.Z.); h18834560910@163.com (X.H.); wfm15090126602@163.com (F.W.)

**Keywords:** myocardial infarction, S100a8, S100a9, autophagy, apoptosis

## Abstract

Myocardial infarction (MI), a type of coronary heart disease, has had a significantly increased incidence in recent years. The balance of cardiomyocyte apoptosis and autophagy after MI is one of the main determinants of patient prognosis. Both affect myocardial fibrosis and ventricular remodeling and regulate cell survival. However, there are few studies on the regulation mechanism of cardiomyocyte autophagy and apoptosis in the early stage after MI. In this study, based on analyzing the scRNA-seq and mRNA-seq data of mice in the early stage of MI, we found that the expression of S100a8 and S100a9 increased first and then decreased in the early stage of MI, and their expression level changed with the number of neutrophils. Further, through the functional enrichment analysis of the differentially expressed genes, we found that S100a8 and S100a9 were simultaneously associated with autophagy and apoptosis and could regulate autophagy and apoptosis of cardiomyocytes through MAPK or PI3K-AKT signaling pathways. This study provides valuable insights for clarifying the pathogenesis of early stage MI and improving its early treatment.

## 1. Introduction

Myocardial infarction (MI), a type of coronary heart disease, has had a significantly increased incidence in recent years. Although the early survival rate of acute myocardial infarction (AMI) has been improved greatly with the introduction of direct percutaneous coronary intervention [1], AMI still has a high probability of causing heart failure and even death. After MI, neutrophils invade in large quantities, quickly occupy the lesion site, and express inflammatory cells to form local damage-associated molecular patterns (DAMPs), which cause an endogenous immune response, including increased expression of pro-inflammatory factors, and even cause cell death [2]. Cell death in early MI mainly affects the size of the later infarct and is also a crucial determinant of the patient’s prognosis in MI [3]. In 2018, Hashimoto H. et al. showed that myocardial tissues at the site of cell death are immediately replaced by fibrotic scar tissues, resulting in the loss of myocardial contractility and ultimately leading to heart failure [4]. Therefore, early cell death is crucial for the late changes in the infarcted heart, and the detailed mechanism of the early changes of MI caused by cell death needs to be further explored.

After MI, neutrophils express mass pro-inflammatory factors at the infarct site, rapidly form local inflammation, and eventually induce cell death [5]. There are many pathways involved in cell death after MI. The intrinsic apoptosis pathway, by causing mitochondrial structure disruption through an increase in apoptotic factors, can cause type I death of cardiomyocytes. Additionally, the autophagy pathway can cause type II cell death due to the excessive degradation of intracellular proteins [6]. Both autophagy and apoptosis are natural physiological processes. Autophagy is a physiological mechanism that relies on lysosomes to degrade metabolic waste and dead tissues and perform energy conversion to provide nutrients for other biological processes. Apoptosis is a process of programmed cell death that cleans up debris in damaged tissue [7,8,9]. The relationship between autophagy and apoptosis is complex, but they have some same molecular signals, and their regulatory pathways are also interrelated and crossed. The balance between autophagy and apoptosis can affect cell survival issues to a large extent [10]. In recent years, many reports have demonstrated that autophagy and apoptosis were induced during MI, but their specific effects and consequences have not yet been unified [11,12,13], especially in the early stage of MI. Some studies have shown that both are beneficial to protecting heart function and promoting benign heart remodeling, but they also can cause cell death [14]. There are still great controversies about the regulatory pathways and molecular mechanisms of autophagy and apoptosis in heart disease.

In tumors and other chronic inflammatory diseases, certain inflammatory factors (such as S100a8 and S100a9) are closely related to autophagy and apoptosis and can regulate cell migration and survival through the mTOR signaling pathway or NF-κB signaling pathway to affect the metastasis of tumor cells [15,16,17]. At the same time, the S100a8/a9 changes in the early stage of MI could affect the infarcted area by controlling the secretion of inflammatory factors and neutrophil migration. In order to further clarify the detailed mechanism caused by S100a8 and S100a9 in the early stage of MI, we conducted an in-depth bioinformatics analysis on two datasets (GSE135310 and GSE151834). The expression patterns of S100a8 and S100a9 in the early stage of MI were obtained, and the gene ontology (GO) and Kyoto Encyclopedia of Genes and Genomes (KEGG) analysis were conducted to assess the potential connections between the genes of S100a8 and S100a9 and autophagy as well as apoptosis. We also revealed the crucial signaling pathways related to autophagy and apoptosis in the early stage of MI.

## 2. Materials and Methods

### 2.1. Data Sources

In this study, two datasets, including GSE135310 and GSE151834, were downloaded from the GEO database (http://www.ncbi.nlm.nih.gov/geo/, accessed on 31 May 2021). GSE135310 is based on the GPL24247 Illumina NovaSeq 6000 (*Mus musculus*). They are single-cell RNA sequencing Cellranger standard files for cardiac CD45+ total leukocytes, which were extracted from the heart or blood of mice in a steady state and at various time points after MI or sham surgery. It consists of 7 sets of samples, and we analyzed 4 of them, including F8 (steady state), F9 (day 7), F11 (day 1), and G1 (day 3). GSE151834 was based on the GPL21103 Illumina HiSeq 4000 (*Mus musculus*). In this dataset, hearts were harvested at 3, 7, 14, 21, and 42 days after ischemic injury, and the scar tissue in the injured region and uninjured region was dissected from the same heart for RNA sequencing. Its expression value was normalized through the ‘limma’ package (version 3.46.0) in R software (version 4.0.3, https://www.r-project.org/, accessed on 19 November 2020).

### 2.2. Identification of Differentially Expressed Genes (DEGs)

GSE135310 is composed of Cellranger standard files of different time points, so we use the ‘Matrix’ package (version 1.3.4) to convert it into counts data format. The converted counts were normalized by CPM using the TMM method, ‘edgeR’ package (version 3.32.1) was further applied to identify DEGs, and we chose |log2 fold change (FC)| ≥ 1 and adjusted *p* value < 0.05 as the screening criteria. GSE151834 is RPKM data. We used the ‘Limma’ package to convert it to TPM and then identified DEGs with the same criteria as before. Finally, the ‘ggplot2’ package (version 3.3.5) was applied to establish volcano plots to show the difference in the genes between the normal group and the disease group in above two datasets.

### 2.3. Cell Cluster Analysis

To observe the expression of different cell types, the ‘Seurat’ package (version 4.0.2) was adopted in R software. We created seurat objects for the data GSE135310, then performed tSNE cluster analysis on it after normalization and discovered the marker genes of each cluster. We also defined the types of clusters by comparing them with the maker genes on the website (https://panglaodb.se/search.html and biocc.hrbmu.edu.cn/CellMarker, accessed on 16 July 2021).

### 2.4. Functional Enrichment Analysis of DEGs

We carried out three methods to annotate and analyze the biological characteristics of these DEGs, including the GO analysis, KEGG pathway analysis, and Gene Set Enrichment Analysis (GSEA). In R software, ‘clusterProfiler’ package (version 3.18.1) was employed to execute GO and KEGG analysis of the selected differential genes. In this analysis, symbol codes were converted to Entrez ID using Mouse genome annotation package ‘org.Mm.eg.db.’ (version 3.12.0), and the adjusted *p* value < 0.05 and Q value < 0.05 are thresholds. ‘fgsea’ (version 1.16.0) and ‘tidyverse’ (version 1.3.1) packages were used to carry out GSEA analysis of all differential genes. The ‘reactome.db’ was chosen as the reference gene set. Gene set permutations were performed 100,000 times for each analysis. The cut-off point of significance was adjusted *p* value < 0.01 for GSEA. Finally, we visualized all the enrichment results through ‘ggplot2’ (version 3.3.5), ‘enrichplot’ (version 1.10.2), and ‘Goplot’ (version 1.0.2) packages.

### 2.5. Protein–Protein Interaction Analysis

We utilized ‘BiocManager’ (version 1.30.16) and ‘STRINGdb’ (version 2.2.2) packages in the R software to perform PPI analysis to construct a network of protein–protein interactions.

## 3. Result

### 3.1. The Schematic Diagram of the Study

**Figure 1:**
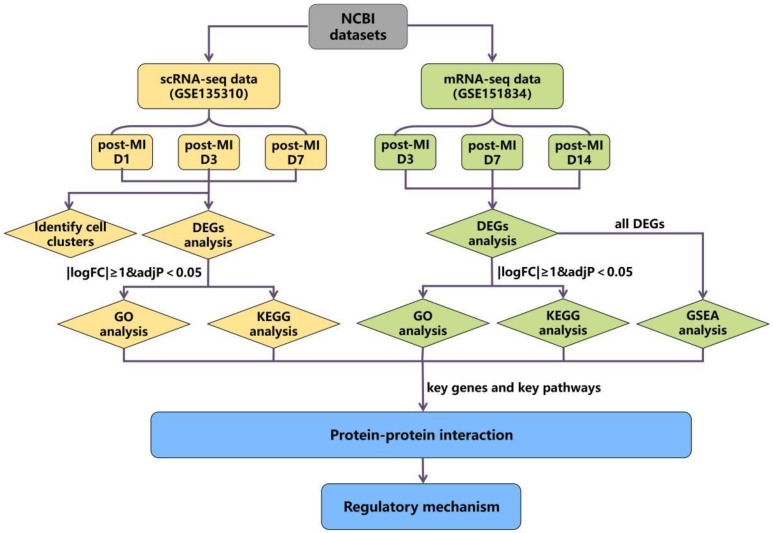
The flow diagram of data analysis.

The schematic diagram of the data analysis is shown in Figure 1. We conducted bioinformatics analysis in two parts; one is the scRNA-seq dataset (GSE135310): Vafadarnejad E. et al. extracted CD45+ total leukocytes through Fluorescence-activated Cell Sorting (FACS) from the heart of mice in the steady state (day 0), 1, 3, and 7 days post-MI, and the genes expressions of each cell were obtained by single-cell RNA sequencing [18]. The other part involved mRNA-seq datasets (GSE151834): the injury sites and uninjured parts from the same heart in 3, 7, 14, 21, and 42 days after ischemic injury were collected separately [19] and used for mRNA sequencing to quantify the temporal changes in gene expression.

Firstly, we performed cluster analysis on scRNA-seq datasets to observe the expression of key genes in various types of cells. Then, differential expression analysis was performed on both two datasets, respectively, to compare whether the genes in the two datasets that meet the |logFC| ≥ 1 & adj *p* < 0.05 standard overlapped or not. The genes that met |logFC| ≥ 1 & adj *p* < 0.05 were screened as DEGs, and we focused on the analysis of genes that were significantly differentially expressed in both two datasets, such as S100a8 and S100a9. Next, we applied enrichment analysis on the DEGs of the two datasets. Since the expression changes of key genes decreased over time and were not screened as DEGs in the later stage, we carried out GSEA analysis on all differential genes at each time point in the mRNA-seq datasets. Through the above analysis, we found the key genes and pathways that are potentially related to cardiac cell autophagy and apoptosis. Finally, we adopted PPI analysis on the above key genes to find the interaction relationship between genes and pathways, and a final mechanism network diagram was constructed.

### 3.2. The Expression Patterns of S100a8 and S100a9 in Neutrophils in the Early Stage of MI Using scRNA-seq

**Figure 2:**
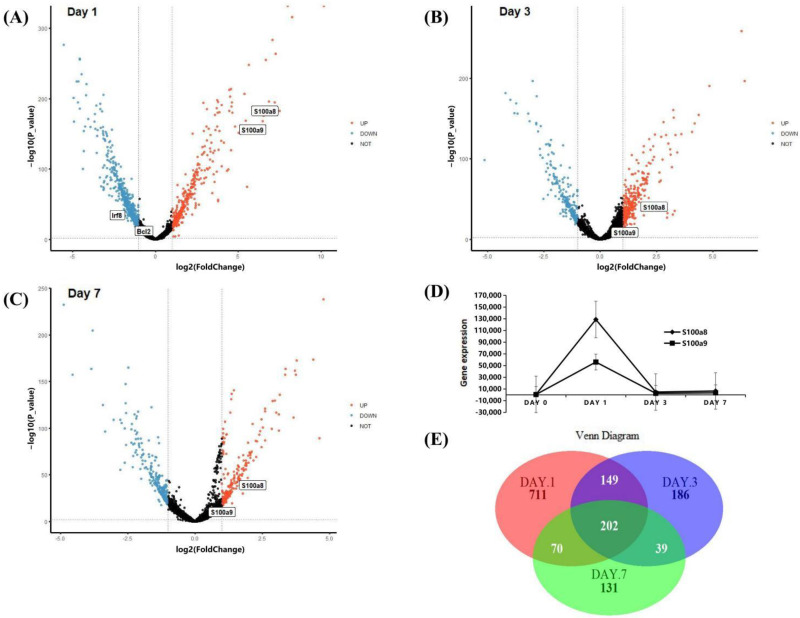
DEGs analysis of scRNA-seq data. (**A**) Volcano plot of changes in CD45+ total leukocyte gene expression in the heart on day 1 post-MI; and (**B**) for day 3 post-MI; (**C**) for day 7 post-MI. Red dots denote genes with log2 FC > 1 and *p* < 0.05; blue dots denote genes with log2 FC < −1 and *p* < 0.05. (**D**) Line chart of S100a8 and S100a9 expression change after MI in the scRNA-seq data. The diamond line represents S100a8, and the square line represents S100a9. (**E**) Venn diagrams of significant DEGs detected in infarct group vs. normal group at 1, 3, and 7 days post-MI.

The scRNA-seq data were obtained from CD45+ cells sorted by flow cytometry in the right ventricular myocardial tissue of MI mice. The ‘TMM’ method was used to standardize the data in R software, we detected 27,998 genes at each time point, and the different gene comparisons of each group are shown in Figure 2A–C. On day 1 post-MI, a total of 1132 DEGs were discovered, which was the day with the largest and most significant gene differential expression in the three time points. Compared with the normal group, 355 genes were up-regulated, and 777 genes were down-regulated. We found that S100a8 and S100a9 were up-regulated very significantly (Figure 2A). On day 3 post-MI, 333 of 575 DEGs were up-regulated, and the other 242 genes were down-regulated (Figure 2B). On day 7, there were fewer DEGs, only 441, of which 198 genes were up-regulated, and 243 genes were down-regulated (Figure 2C). On the 3rd and 7th day, S100a8 and S100a9 were slightly increased. The overall change trend of S100a8 and S100a9 was to increase first but then slowly decrease and reach the peak on the first day. Furthermore, the change of S100a8 was more obvious in the right ventricle of mice (Figure 2D). There are 202 genes, including S100a8, with significant changes at three time points (Figure 2E). S100a8 and S100a9 are mainly expressed in the liver, spleen, or cancer diseases [20]. In 2008, they were experimentally proved to be up-regulated in lipopolysaccharide-induced heart disease for the first time [21]. In addition to myocardial tissue, S100a8 and S100a9 are also highly expressed in the blood of patients with acute myocardial infarction [22]. In this study, we noticed that S100a8 and S100a9 were significantly changed in the right ventricle of MI mice, so we focused on the key role of S100a8 and S100a9 in the early stage of MI in follow-up research.

**Figure 3:**
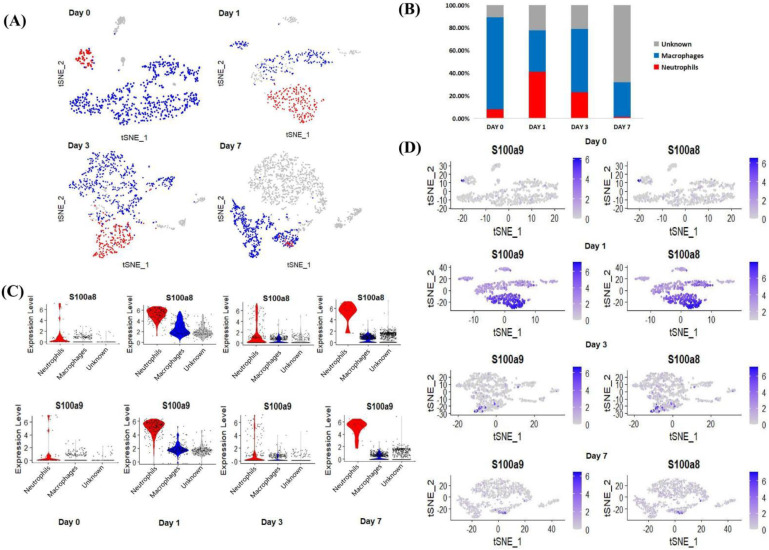
Cluster analysis of scRNA-seq data. (**A**) The classification of cardiac CD45+ total leukocytes from day 0 to day 7, using t-distributed random neighbor embedding, and coloring by clustering after manual annotation. Blue (macrophages), red (neutrophils), gray (unknown). (**B**) The proportion of neutrophils, macrophages and unknown clusters among the total number of cells at each time point. (**C**) Violin diagram of the expression of S100a8 and S100a9 in various types of cells at each time point. (**D**) S100a8 and S100a9 expression distribution map at each time point.

After normalization and standardization of the obtained scRNA-seq data, a total of 666 cells were obtained on day 0, 528 cells on day 1, 943 cells on day 3, and 1796 cells on day 7, with 27,998 genes per cell (Figure 3A). Subsequently, we grouped daily cells based on gene expression profiles, and t-distributed stochastic neighbor embedding (t-SNE) plots were taken for visualization. According to the expression of neutrophil markers (Hp, G0s2) and macrophage markers (Cd68, Il1b, Lyz1, Cd74) [23,24], we divided the cells at each time point into three categories: neutrophils (red), macrophages (blue), and unknown (gray). After MI, the number of neutrophils increased greatly and reached a peak on day 1 post-MI, accounting for 41.10% of the total number of CD45+ cells (Figure 3B). After the third day, neutrophils gradually decreased, and other cells, such as T/B lymphocytes, NK cells, and endothelial cells, increased conversely, making up 68.10% (Figure 3B). It is worth noting that the change in the proportion of neutrophils was similar to the change in the expression level of S100a8 and S100a9.

Next, we visualized the expression of the key genes in the above three types of cells. From the above analysis, before MI (day 0), S100a8 and S100a9 were almost exclusively expressed in neutrophils, and with the development of MI (day 1–day 7), the expression of both factors increased, mainly in neutrophils (Figure 3C). Since our data showed that the expression of S100a8 and S100a9 and the number of neutrophils increased in tandem, we demonstrated from the level of scRNA-seq detection that S100a8 and S100a9 invading the infarct site after MI, including a large number of other inflammatory factors, may be mainly derived from newly born neutrophils (Figure 3D).

### 3.3. S100a8 and S100a9 Are Significantly Enriched in the Biological Processes of Autophagy and Apoptosis by scRNA-seq

**Figure 4:**
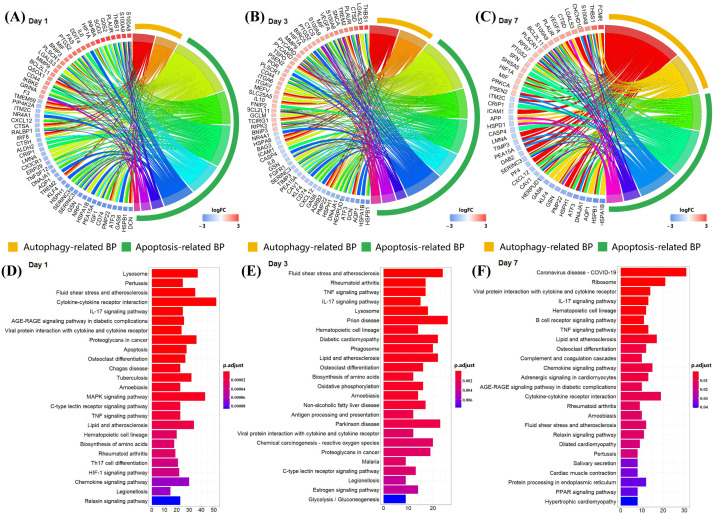
Enrichment analysis of DEGs at each time point in scRNA-seq data. (**A**) Circle diagram of the biological process analysis of significant DEGs on day 1 post-MI and (**B**) for day 3 post-MI, (**C**) for day 7 post-MI. Different colored blocks represent different biological processes. The yellow band in the outermost circle indicates autophagy-related BPs, and the green band indicates apoptosis-related BPs. (**D**) Bar chart of the KEGG analysis of significant DEGs on day 1 post-MI and (**E**) for day 3 post-MI, (**F**) for day 7 post-MI. Only the first 25 pathways are shown in the figure, and the *p* cut-off value of all pathways is 0.05.

In order to further understand the role of S100a8 and S100a9 in the recovery of MI, we performed GO and KEGG analyses of DEGs at each time point and selected *p* < 0.05 biological processes and pathways. The GO analysis showed that the gene expression changes in CD45+ leukocytes after MI were mostly related to cell adhesion, myeloid cell migration, cell chemotaxis, and inflammation response (Figure S1A–C). Like the previous report, S100a8 and S100a9 were also mainly enriched in the above four biological processes. To our surprise, apart from the above processes, they were also enriched in the biological processes related to autophagy and apoptosis (Figure 4A–C). In all three time points, S100a8 and S100a9 were annotated in autophagy-related BPs, including autophagy (GO:0006914) and a process utilizing the autophagic mechanism (GO:0061919); meanwhile, they were also annotated in the regulation of the apoptotic signaling pathway (GO:2001233), the regulation of the intrinsic apoptotic signaling pathway (GO:2001242), and other apoptosis-related BPs. Previously, the involvement of S100a8/a9 in autophagy and apoptosis was found in various tumors and other chronic inflammatory diseases [6,25], but that was rarely mentioned in cardiovascular diseases. However, the KEGG pathway analysis demonstrated that S100a8 and S100a9 were merely related to the ‘IL-17 signaling pathway’ (mmu04657) in all time points (Figure 4D–F). In addition, ‘lysosomes’, ‘apoptosis’, and ‘MAPK signaling pathways’ are significantly enriched. All enrichment results are shown in the Supplementary Information. After consulting the literature, we found that in psoriasis and cancer diseases, the IL-17 signaling pathway can cause the production of pro-inflammatory factors such as S100a8 and S100a9, thereby promoting the generation of inflammation and the metastasis of cancer cells [26,27]. So, we speculate that this pathway may also be applicable in heart tissue, causing the autophagy and apoptosis of heart cells. Moreover, we realized that S100a8 and S100a9 mostly work through exocrine, so we obtained the mRNA-seq data of the same heart injury and uninjured parts at different time points after MI, focusing on the analysis of the relationship between S100a8, S100a9, and autophagy and apoptosis.

### 3.4. The Expression Patterns of S100a8 and S100a9 in the Early Stage of MI Using mRNA-seq

**Figure 5:**
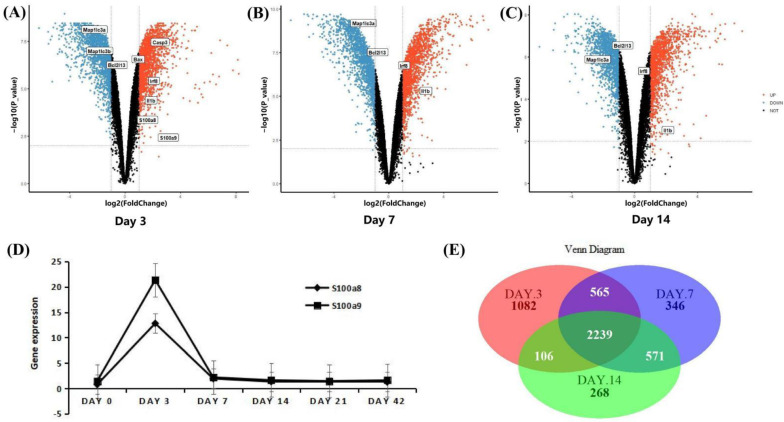
DEGs analysis of mRNA-seq data. (**A**) Volcano map of the difference in gene expression between injured and uninjured areas of the same heart on day 3 post-MI; and (**B**) for day 7 post-MI; (**C**) for day 14 post-MI. The filter conditions are the same as above: |log2 FC| > 1 and *p* < 0.05. (**D**) Line chart of S100a8 and S100a9 expression change after MI in the mRNA-seq data. (**E**) Venn diagrams of significant DEGs detected in injured vs. uninjured areas at 3, 7, and 14 days post-MI.

The mRNA-seq data were derived from infarcted and non-infarcted regions of the whole mouse heart. In order to improve the comparability between different data indicators, we first normalized the expression values of the data for 3, 7, and 14 days, so that the different indicators are in the same order of magnitude (Figure S2A–C). There are four uninjured groups and four injured groups every day. A total of 3992 DEGs were detected on the third day, of which 1999 were up-regulated, including S100a8 and S100a9 genes, and the rest were down-regulated (Figure 5A). On day 7 post-MI, there were 3720 DEGs, of which 1866 were up-regulated, and 1854 genes were down-regulated (Figure 5B); on day 14 post-MI, there were 3183 DEGs, including 1553 up-regulated and 1630 down-regulated (Figure 5C). Consistent with the above scRNA-seq data, the expressions of S100a8 and S100a9 first increased and then decreased, but the difference is that scRNA-seq showed that in the right ventricle, the change of S100a8 was greater than that of S100a9; but mRNA-seq showed that from the whole heart, the change of S100a9 was more significant (Figure 5D). A total of 2239 genes changed simultaneously in three days (Figure 5E). Furthermore, we also visualized the marker genes of inflammation, autophagy, and apoptosis in the volcano maps. Inflammation marker genes Irf8 and Il-1β were significantly up-regulated; the autophagy marker gene Map1lc3 decreased remarkably; and Casp3 and Bax, apoptotic marker genes, increased significantly (Figure 5A–C).

### 3.5. S100a8 and S100a9 Are Involved in Signaling Pathways Related to Rho GTPases by mRNA-seq

**Figure 6:**
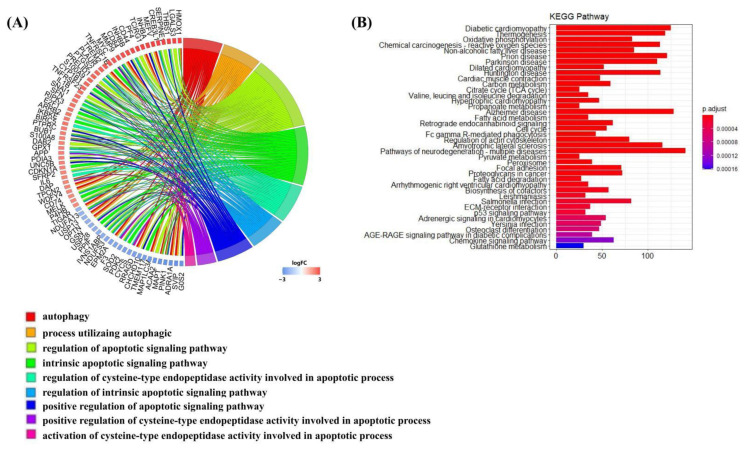
Enrichment analysis of DEGs on day 3 post-MI in mRNA-seq data. (**A**) Circle diagram of the biological analysis of DEGs on day 3 post-MI. Different color blocks represent different biological processes. The circle diagram below shows the biological processes associated with autophagy and apoptosis. (**B**) Bar chart of the KEGG analysis of significant DEGs on day 3 post-MI. The first 40 pathways are shown in the figure, and the *p* cut-off value of all pathways is 0.05.

The R software was employed again for the GO and KEGG enrichment analysis of DEGs at each time point of the mRNA-seq data, and the screening conditions were the same as that of scRNA-seq. In this analysis, since S100a8 and S100a9 were not included as DEGs due to their adjusted *p* values > 0.05 on day 7 and day 14, S100a8 and S100a9 were only annotated to the biological processes connected to autophagy and apoptosis on day 3 post-MI by GO analysis (Figure 6A). KEGG analysis indicated that DEGs were mostly enriched in oxidative metabolism, cardiomyopathy, and cell circulation (Figure 6B). They were also involved in the ‘Il-17 signaling pathway’, but its adj *p* > 0.05.

**Figure 7:**
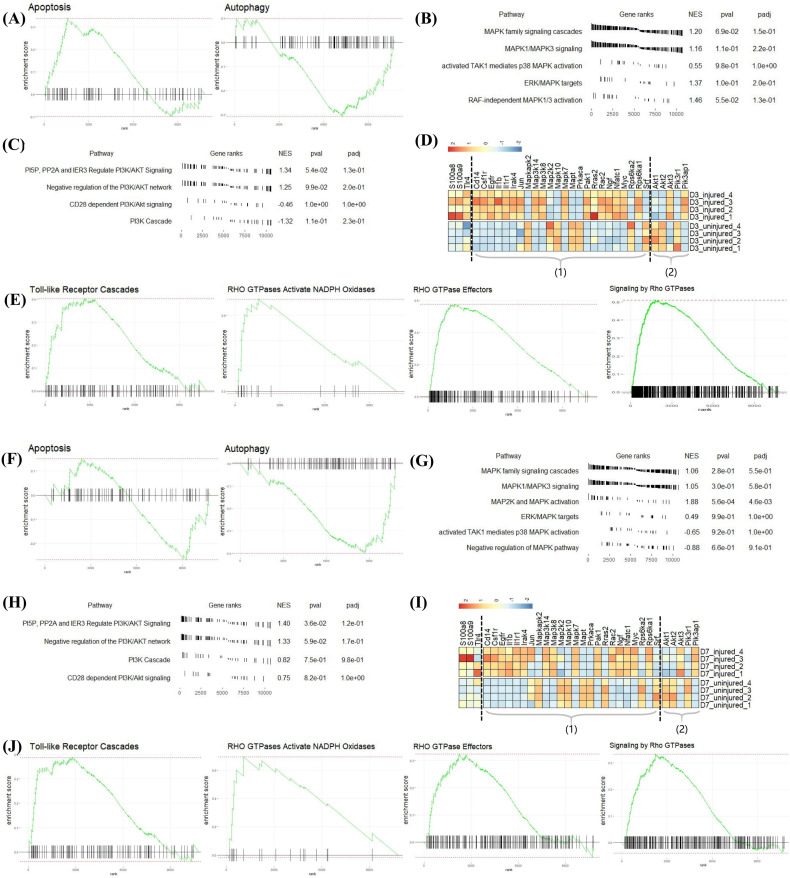
GSEA analysis of all DEGs at each point post-MI in mRNA-seq data. (**A**) Apoptosis and autophagy enriched in DEGs on day 3 post-MI, (**F**) day 7 post-MI. (**B**,**C**), respectively, represent the expression of related items in the MAPK signaling pathway and PI3K-AKT signaling pathway on day 3 post-MI; (**G**,**H**) are on day 7 post-MI. (**D**,**I**) Heatmaps revealing the expression of key genes in the injured and uninjured areas at various time points after MI. Part (1) includes MAPK-signaling-pathway-related genes; (2) contains PI3K-AKT-signaling-pathway-related genes. (**E**,**J**) 4 GSEA pathways annotated by S100a8/a9 on day 3 post-MI. NES: Normalized Enrichment Score, which normalizes the enrichment score according to the size of the gene set.

The GO analysis of the above two datasets shows that S100a8 and S100a9 were involved in the autophagy and apoptosis of heart cells, but the specific regulation pathway was still unclear. Therefore, we performed GSEA analysis on all differentially expressed genes at each time point. By comparing with the ‘reactome.db’ gene set, we found that on day 3 post-MI, autophagy (NES = −1.21, padj = 0.24) was significantly inhibited, and apoptosis (NES = 1.35, padj = 0.11) was significantly promoted (Figure 7A), consistent with changes in the expression of related genes shown in Figure 5A. On day 7 post-MI, autophagy (NES = −1.52, padj = 0.03) was still suppressed, and apoptosis (NES = −0.98, padj = 0.77) changed from being increased to being reduced (Figure 7F). At the same time, the related entries of the MAPK signaling pathway and the PI3K-AKT signaling pathway, which were reported to regulate autophagy and apoptosis, were also enriched on day 3 and day 7 post-MI (Figure 7B,C,G,H). The gene expression changes associated with the above two pathways are shown in the volcano map of Figure 7D (day 3) and Figure 7I (day 7). Additionally, S100a8 and S100a9 were mainly annotated in the four pathways of ‘Toll-like Receptor Cascades’, ‘RHO GTPases Activate NADPH Oxidases’, ‘RHO GTPase Effectors’, and ‘Signaling by Rho GTPases’ at the three time points (Figure 7E,J). In the light of literature, TLR4 is the receptor of S100a8 and S100a9, and they can regulate many biological functions through the Toll-like Receptor Cascades response, such as releasing and regulating inflammatory factors [28,29]. Extensive literature has reported that Rho GTPases have a high connection with the cell-survival-related MAPK signaling pathway and PI3K-AKT signaling pathway. Consequently, we supposed that S100a8 and S100a9 might regulate cardiac cell survival problems through Rho GTPases.

### 3.6. PPI Network Predictions of S100a8/a9 and Genes Related to the MAPK Signaling Pathway and PI3K-AKT Signaling Pathway

**Figure 8:**
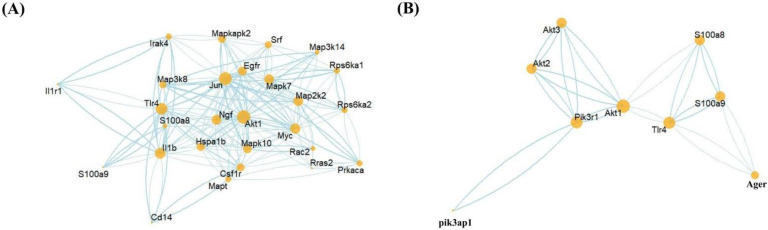
Protein–Protein Interaction Networks (PPI) prediction. The PPI network between the MAPK-signaling-pathway-related genes (**A**); PI3K-AKT-signaling-pathway-related genes (**B**); and S100a8, S100a9, and their receptor TLR4. The node size represents the connectivity degree, and the closer to center, the more critical it is.

To further confirm the relationship between S100a8, S100a9, and the MAPK pathway and PI3K-AKT pathway, we linked the STRING database through R software to check the potential protein interaction relationship between these genes and generated two PPI networks (Figure 8). Multiple MAPK-pathway-related genes, including Mapk7, Mapk10, and Mapkapk2, interacted with S100a8, S100a9, and their receptor TLR4 (Figure 8A). The PPI network of genes related to S100a8 and S100a9 and the MAPK signaling pathway contained 32 nodes and 457 edges. In contrast, there were fewer genes concerning the PI3K-AKT signaling pathway, but they are also tightly linked with S100a8 and S100a9; this PPI network had 9 nodes and 16 edges (Figure 8B). The above-mentioned genes were remarkably up-regulated or down-regulated at different time points after MI, which was consistent with the previous KEGG analysis. In summary, we rationally speculated that S100a8 and S100a9 regulate the autophagy and apoptosis of cardiac cells in the early stage of MI through the MAPK signaling pathway and PI3K-AKT signaling pathway.

### 3.7. The Possible Mechanism of S100a8 and S100a9 in Regulating Cardiac Cells Autophagy and Apoptosis in the Early Stage of MI

**Figure 9:**
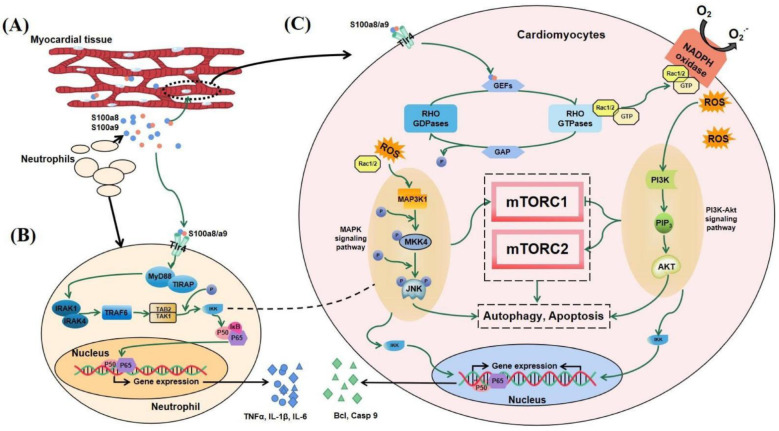
Possible mechanism diagram of S100a8/a9 regulating autophagy and apoptosis in the early stage of MI. (**A**) Neutrophils transfer to the injured area of MI and release S100a8/a9. Part of S100a8/a9 returns to neutrophils to regulate gene expression (**B**), and the other part enters cardiomyocytes to regulate cell death (**C**).

Combining the above results and published literature, we found that S100a8 and S100a9 may regulate the autophagy and apoptosis of cardiomyocytes through multiple interlocking signaling pathways in the early stage of MI. Within one day of cardiac infarction, newborn neutrophils accumulate in large numbers at the infarct area, releasing dangerous signal factors such as S100a8 and S100a9 (Figure 9A). As a kind of DAMPs, S100a8/a9 partly returns to neutrophils to regulate DNA transcription through the NF-κB signaling pathway, allowing cells to express and release IL-1β, TNF-α, and other pro-inflammatory factors (Figure 9B). The other part enters cardiomyocytes or other heart cells through TLR4 and combines with GMP exchange factors (GEFs) to convert Rac1/2 into a viable form of Rho GTPases, and then Rac1/2 activates the MAPK signaling pathway and the PI3K-AKT signaling pathway. The above two pathways can not only directly regulate cell survival but also indirectly regulate autophagy and apoptosis through the mTOR signaling pathway. In addition, through the NF-κB signaling pathway, these two pathways can also activate the expression of some factors, such as Bcl and Casp9, which are related to apoptosis and autophagy (Figure 9C).

## 4. Discussion

Understanding the early changes of MI can help us find better ways to control the progression of MI. In this study, the bioinformatics method was used to analyze two groups of data, which were both detected in the early stage of MI. Mouse S100a8 and S100a9 proteins, also known as MRP8 and MRP14 (myeloid-related proteins), belong to the S100 protein family, which includes 21 types and have a common Ca^2+^ binding motif, the EF-hand [30]. Our study demonstrated the changing pattern of S100a8 and S100a9 in neutrophils in the early stage of MI. S100a8/a9, called calprotectin, is highly expressed in inflammatory diseases. In cancer or certain chronic inflammatory diseases, it acts as a DAMP molecule; binds with TLR4, RAGE, and calcium ions; and regulates biological processes, such as cell proliferation and cell migration [31,32,33]. Previous experiments showed that the expression of S100a8/a9 is significantly increased in MI [18,19]. However, the origin of S100a8/a9 needs to be further clarified, and the role of changes in S100a8/a9 at different stages after MI remains to be studied.

Cardiac repair after MI is divided into three stages [34], which coincide with the expression changes of S100a8 and S100a9 analyzed in this study. The first stage is the acute inflammation phase (~day 1): necrotic cardiomyocytes (CMs) release danger signals, which quickly activate the resident sentinel cells, thereby initiating the recruitment of neutrophils to remove cell debris [35]. In the analyzed data, the levels of S100a8 and S100a9 and the number of neutrophils were increased significantly at the infarcted site during this period. After S100a8 and S100a9 are expressed, respectively, driven by intracellular Ca^2+^, they combine with each other to form a heterodimeric EF-hand Ca^2+^ binding protein, calprotectin, thereby transmitting Ca^2+^ information and participating in subsequent pathways. The changes in lysosome and apoptotic pathways were the most significant on day 1 after MI by GO and KEGG analysis. The second stage is the subacute proliferation stage (~day 3): cardiac fibroblasts (CFs) secrete large amounts of collagen and matrix proteins to compensate for cell loss [36,37]. The third stage is mature stage (~day 7): the collagen secreted in the previous stage is enzymatically cross-linked, the heart remodeling is gradually completed, and the cell state tends to be stable [36,37]. In the latter two stages, the increase in S100a8 and S100a9 expression tended to be flat, and the biological processes of collagen formation and cell adhesion gradually became prominent. S100a8 and S100a9 can also cause stent thrombosis in the very late stage of acute myocardial infarction [38]. The mRNA expression of S100a8 and S100a9 obtained in this study was derived from the right ventricle and whole heart tissue. However, the mRNA expression of S100a8 and S100a9 in the corresponding tissues of different diseases may lead to changes in the expression of proteins during translation, thus becoming one of the important causes of certain diseases.

From the cell cluster analysis, we can note that almost all S100a8 and S100a9 were from neutrophils (Figure 3A,D). Researchers reported that neutrophils are the main source of S100a8/a9, which have become the treatment targets of cardiac inflammation and the following damage [39,40,41]. Using scRNA sequencing, we found that S100a8, S100a9, and neutrophils changed in synchrony, further elucidating that S100a8 and S100a9 were derived from neonatal neutrophils in the early stage of MI rather than resident neutrophils. Furthermore, we believe that the change in the proportion of neutrophils is related to the leukocyte lifespan, which is usually only 3–7 days [35,42], so neutrophils show a trend of increasing first and then decreasing after MI.

Through enrichment analysis, we were surprised to find that S100a8 and S100a9 are closely related to autophagy and apoptosis in the early stage of MI. Autophagy and apoptosis are considered to be decisive pathways leading to various human diseases, such as cancer, inflammatory diseases, heart failure, and the aging process [43]. At the same time, the pathway analysis prompted us to find four vital signaling pathways that may be related to autophagy and apoptosis. The first two pathways were the ‘MAPK signaling pathway’ and the ‘PI3K-AKT signaling pathway’, which were both prominent in the KEGG analysis of scRNA-seq and the GSEA analysis of mRNA-seq. From the results of the GSEA analysis, we found that these two signaling pathways were disturbed to varying degrees in the early stage post-MI. As early as 2006, Elisabeth Corcelle et al. experimentally proved that in tumor suppression, activating the mitochondrial MAPK-ERK signaling pathway can corrupt autophagy [44]. In 2018, the MAPK signaling pathway regulating cell activity was once again verified in even more detail in inflammatory diseases such as colitis [45]. On the other hand, a large number of experiments found that the PI3K-AKT signaling pathway is one of the important ways to control cell autophagy and apoptosis and is involved in initiating and promoting a series of pathological diseases, including various tumors [46]. The PI3K-AKT-mTOR-autophagy signal axis has also become the preferred treatment strategy for various tumors and inflammatory diseases [47]. However, the roles of the above two signaling pathways in MI have not been discovered. The third pathway is the Rho GTPases-related signaling pathway. In 2019, Xi Zhang et al. found that matrine slows the signal transduction of PI3K-AKT and mTOR signaling pathways by reducing the phosphorylation level of RhoA, a Rho GTPases subtype, thereby regulating autophagy and apoptosis of ovarian cancer cells [48]. Rac1 is another subtype of Rho GTPases that can trigger three MAPK signaling pathways, including JNK, p38, and ERK signaling pathways [49]. We found that S100a8 and S100a9 were directly enriched in signaling pathways associated with Rho GTPases-related signaling pathways by GSEA analysis. Therefore, it is reasonable to speculate that in heart tissue, S100a8 and S100a9 can also participate in MAPK and PI3K-AKT signaling pathways through Rho GTPases-related signaling pathways, thereby regulating cardiac cell autophagy and apoptosis. The fourth pathway is the ’IL-17 signaling pathway’, which is the only pathway explicitly involved in S100a8 and S100a9 in the KEGG analysis of both scRNA-seq and mRNA-seq data. Most studies have shown that in tumors and inflammatory diseases, such as in children with pneumonia, cell damage or other inflammatory factors such as IL-17A can activate the expression of S100a8/a9 through the IL-17 signaling pathway. However, several pieces of research have also illustrated that S100a8/a9 is upstream of the IL-17 signaling pathway, such as in acute pancreatitis. We speculate that the relationship between S100a8/a9 and the IL-17 signaling pathway may depend on the cell type and S100a8/a9 concentration.

So far, Raluca Maria Boteanu et al. found that the expression of S100a8 and S100a9 increased after MI, and short-term use of an S100a9 blocker can reduce the expression of apoptosis-related proteins in MI mouse models, which is beneficial to cardiac recovery after MI [50]. However, long-term S100a9 blockade is detrimental to cardiac repair [51]. Consistent with our results, on day 3 post-MI, the expression level of S100a8 and S100a9 were significantly increased, while on day 7 post-MI, the increase in S100a8 was not obvious; only S100a9 was defined as DEGs. This indicates that S100a9 appears to play a dominant role in the regulation of cardiac remodeling after MI by calprotectin. In addition to apoptosis, we also found that S100a8 and S100a9 are enriched in autophagy-related pathways. It is known that the regulatory pathways of autophagy and apoptosis overlap greatly. We believe that S100a8 and S100a9 may be involved in regulating the balance of autophagy and apoptosis, thereby affecting cell death. In order to further verify the pathways we predicted before, PPI analysis was adopted. Among the genes related to the MAPK signaling pathway, except for S100a8 and S100a9, 10 genes were directly related to TLR4, and their combined scores were all higher than 0.7. The most prominent of these is CD14 (0.999), which is located upstream of the Rac1-p38 MAPK pathway and regulates gene expression in neonatal rat cardiomyocytes [52]. Additionally, among the genes concerning the PI3K-AKT signaling pathway, the most closely related to TLR4 is Pik3r1 (0.852). Pik3r1 is a marker gene of the PI3K-AKT signaling pathway, which directly participates in the pathway and regulates the phosphorylation of the downstream target mTOR [53]. These findings demonstrate the interaction between S100a8, S100a9, and the MAPK and PI3K-AKT pathways. Although this paper demonstrated from the scRNA-seq level that S100a8 and S100a9 are derived from neonatal neutrophils in the early stage of MI and proposes potential pathways for S100a8 and S100a9 to regulate autophagy and apoptosis, this study has some limitations. Bioinformatics combines multiple disciplines to discover the potential mechanism of early stage MI, but the mechanism obtained requires experimental verification in the future. With the rapid development of bioinformatics analysis methods and dimensions, we may obtain more detailed mechanisms of S100a8 and S100a9 involved in the early stage of MI. For example, subsequent more precise spatial sequencing may capture the exact targets of S100a8 and S100a9.

In summary, our study executed a series of analyses on the scRNA-seq data of CD45+ total leukocytes and the mRNA-seq data of the total cells in the early stage of the mouse MI model. Using scRNA-seq analysis, we confirmed that S100a8 and S100a9 significantly increased in the early stage of MI, and most of them came from neutrophils. Furthermore, the number of neutrophils first increased and then decreased with the change in the recovery period after MI. In addition, our findings demonstrate that S100a8 and S100a9 may regulate cardiomyocyte autophagy and apoptosis through MAPK and PI3K-AKT signaling pathways in the early stage of MI. The proposed molecular pathogenesis mechanism will provide a comprehensive theoretical basis for the early diagnosis and treatment of MI.

## Data Availability

Not applicable.

## Supplementary Materials

The following supporting information can be downloaded at: https://www.mdpi.com/article/10.3390/cells11121911/s1. Figure S1: Gene ontology analysis of DEGs at each time point in scRNA-seq data. Figure S2: mRNA-seq data processing results.
