# Supplementary material for: Integrated Analysis Reveals S100a8/a9 Regulates Autophagy and Apoptosis through the MAPK and PI3K-AKT Signaling Pathway in the Early Stage of Myocardial Infarction
Source: Cells. 2022 Jun 13;11(12):1911. doi: 10.3390/cells11121911 (PMC9221389; doi:10.3390/cells11121911)
Supplement: Supplementary file 1 [file cells-11-01911-s001.zip › cells-1752676-supplementary.pdf]

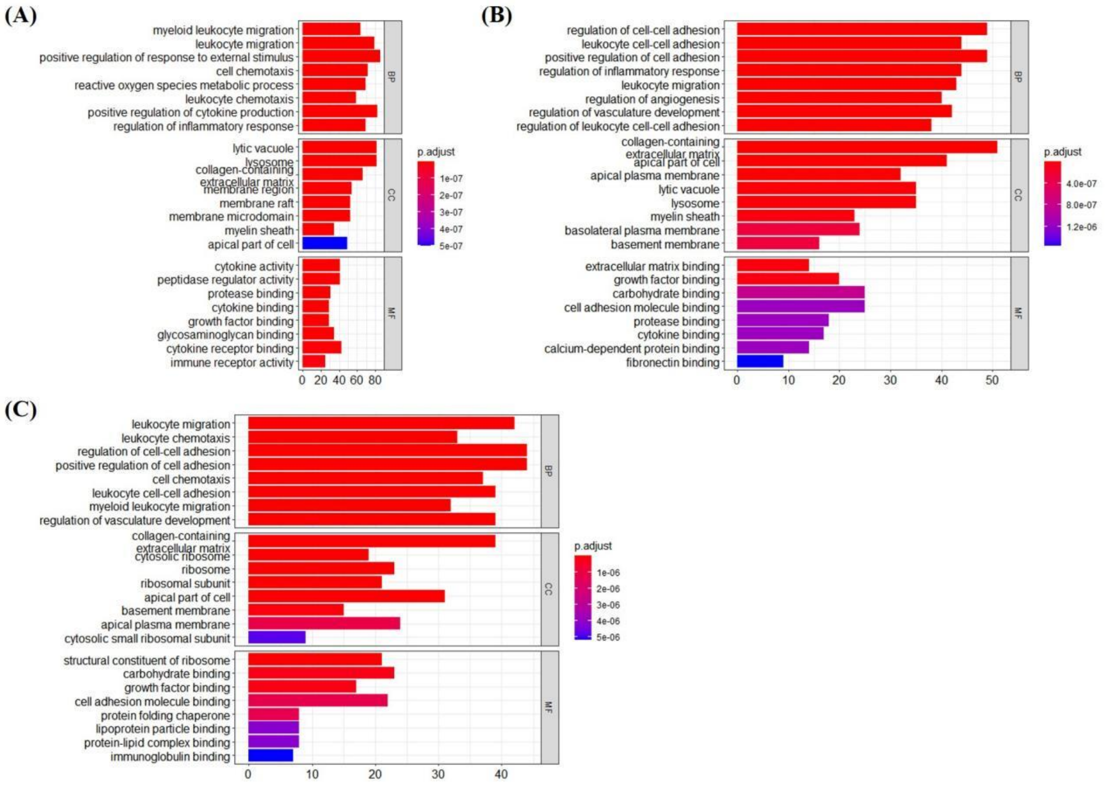

Figure S1: Gene ontology analysis of DEGs at each time point in scRNA-seq data. (A), (B), (C) are bar graphs of GO analysis of significant DEGs on days 1, 3, and 7 post MI, respectively. BP: Biological Process, CC: Cellular Components, MF: Molecular Function, only the first 8 items are displayed in each column.

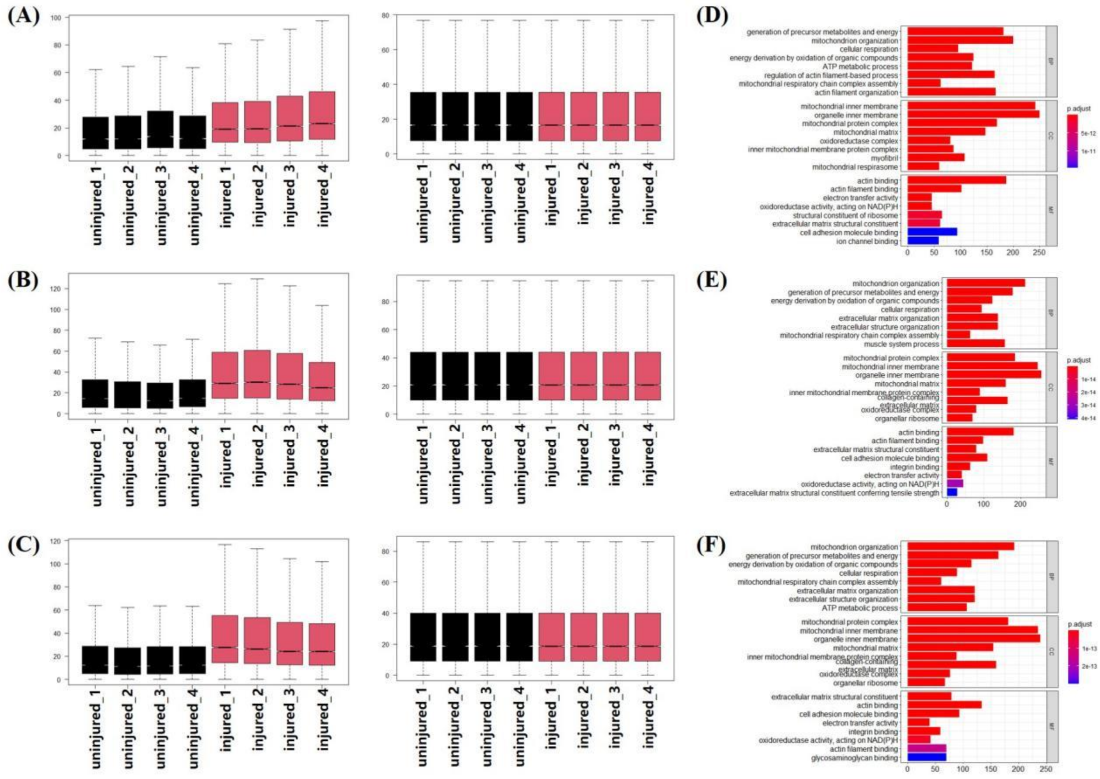

Figure S2: mRNA-seq data processing results. (A) is the comparison before and after normalization of mRNA-seq data on day 3 post MI, (B) represents day 7 post MI, (C) represents day 14 after MI. Black is the control group, red is the MI group, n=4. (D), (E), (F) are bar graphs of GO analysis of significant DEGs on days 3, 7, and 14 post MI, respectively. only the first 8 items are displayed in each column.
